# Impacts of increasing levels of salt on intake, digestion, and rumen fermentation with beef cattle consuming low-quality forages

**DOI:** 10.1093/jas/skae284

**Published:** 2024-09-25

**Authors:** Hayley C White, Noah G Davis, Megan L Van Emon, Hannah M DelCurto-Wyffels, Samuel A Wyffels, Timothy DelCurto

**Affiliations:** Department of Animal and Range Sciences, Montana State University, Bozeman, MT 59717-2900, USA; Department of Animal and Range Sciences, Montana State University, Bozeman, MT 59717-2900, USA; Department of Animal and Range Sciences, Montana State University, Bozeman, MT 59717-2900, USA; Department of Animal and Range Sciences, Montana State University, Bozeman, MT 59717-2900, USA; Department of Animal and Range Sciences, Montana State University, Bozeman, MT 59717-2900, USA; Department of Animal and Range Sciences, Montana State University, Bozeman, MT 59717-2900, USA

**Keywords:** beef cattle, digestibility, intake, rumen fermentation, salt-limited supplement

## Abstract

The objectives of this study were to evaluate the levels of supplemental salt on low-quality forage intake, water intake, dry matter (**DM**) digestibility, and rumen fermentation. Six ruminally cannulated, Angus crossbred heifers (14 mo of age; 449 kg ± 24 kg body weight [**BW**]) were used in a dual 3 × 3 Latin square design. The heifers were housed in individual stalls with 2 animals assigned to each treatment per period. Salt treatments were mixed into a protein supplement of 50% cracked corn and 50% soybean meal and fed at 0.3% of shrunk BW. Salt treatments consisted of 1) control, no salt (CON), 2) 0.05% of BW salt (LOW), and 3) 0.1% of BW salt (HIGH). Chopped, low-quality (CP = 7.4%; NDF = 64.2%), grass hay was used as the base ration and was provided daily at 120% of the average daily intake of the previous 3 d. Each period included a 14-d diet adaptation, 6 d of sample collection, 1 d collection of rumen fluid samples for ruminal and microbial profiles. Individual forage dry matter intake, water intake, and DM digestibility were measured during the 6-d collection period. Rumen pH, ammonia levels, and volatile fatty acid (VFA) concentrations were measured during the 1-d ruminal profile. Rumen DM and liquid fill were determined with a 5-h post-feeding rumen evacuation. Supplemental salt had no influence on forage intake (*P* = 0.19) expressed on a kg/d basis yet tended to decrease linearly (*P* = 0.06) with increasing levels of salt when expressed on a grams/kg BW basis. DM digestibility was not influenced by salt levels (*P *> 0.05), but DM fill tended to increase linearly with increasing salt levels (*P* = 0.06). Water intake and liquid fill, however, increased linearly with increasing levels of salt (*P* < 0.01) with an 18.9% increase in water intake and 17.0% increase in liquid fill compared to control animals. Ruminal pH and ammonia levels both decreased linearly with increasing salt (*P* < 0.01). Acetate concentration and acetate: propionate ratio increased linearly with increasing levels of salt (*P* < 0.01). In contrast, isobutyrate and butyrate concentrations decreased linearly with increasing levels of salt (*P* < 0.01). Our research suggests that increasing levels of salt tends to influence dry matter intake, DM fill, liquid kinetics, and rumen fermentation characteristics. Results from this research provide additional information on how salt-limited supplements may impact beef cattle consuming low-quality forage diets.

## Teaser

Salt is an effective intake regulator in self-fed supplements. However, for beef cattle consuming low-quality forages, high salt diets may result in lower intakes and less efficient rumen fermentation.

## Introduction

Strategic supplementation is often used by beef cattle producers in extensive environments to compensate for seasonal nutrient deficiencies, and self-limited supplements are a common method to enhance the utilization of low-quality forages (DelCurto et al., 2000). However, one of the main limitations of providing self-fed supplements to grazing cattle is the inability to control individual animal consumption of supplement (Bowman and Sowell, 1997). Past studies have evaluated the intake variation of individual supplement consumption and have found that some animals refuse supplements altogether (non-feeders), while others consume excessive amounts (Bowman and Sowell, 1997; Williams et al., 2018; Wyffels et al., 2020). The inability to regulate supplement intake can result in a discrepancy between animal nutrient requirements and nutrient consumption (Bowman and Sowell, 1997).

The most common method to regulate intake of self-fed supplements is the use of salt (Weir and Miller, 1953; Kunkle et al., 2000). Salt (NaCl) is the most common intake limiter because it is readily available, generally safe, and salt level can be modified to achieve desired levels of intake (Kunkle et al. 2000). However, recent research has demonstrated daily individual intake of salt-limited supplement can be highly variable (Williams et al., 2018; Wyffels et al., 2020; White et al., 2022). This high variability of supplement intake between individuals can reduce animal performance and/or decrease profit margins for the producers (Bowman and Sowell, 1997; Williams et al., 2018; Wyffels et al., 2020), however, little is known about the effects of high salt levels on subsequent intake and digestion of low-quality roughages.

Salt is known to have a metabolic effect on osmotic gradient, regulation of water balance, and control of the acid–base balance (NASEM, 2016). While some research has observed increased water consumption and a subsequent increase in ruminal outflow when cattle were fed high levels of salt (Rogers et al., 1982), this research was conducted on cattle fed high-concentrate diets. Therefore, research evaluating the effects of salt intake on liquid kinetics and ruminal parameters specific to beef cattle consuming low-quality forages would expand the understanding of salt utilization in grazing scenarios. Thus, the objectives of this study were to evaluate the impacts of increasing supplemental salt levels on forage intake, water intake, digestibility, and rumen fermentation of beef cattle consuming high-fiber, low-quality forages. We hypothesized that increasing levels of salt modify rumen fermentation characteristics and digestion by increasing water intake and impacting liquid kinetics which in turn can influence forage intake, digestibility, and rumen fermentation.

## Materials and Methods

Experimental procedures described herein were approved by the Agriculture Animal Care and Use Committees of Montana State University (#2017-AA09). All animals used in this study were provided by the Montana Agricultural Experiment Station, and the study was conducted July–September at the Bozeman Agriculture Research and Teaching (BART) Farm at Montana State University in Bozeman, Montana.

Six Angus crossbred heifers (14 mo of age; 377 kg ± 27 kg body weight [**BW**]) were surgically fitted with a ruminal cannula (Bar Diamond, Inc., Parma, ID, USA), housed in individual pens, and randomly assigned to 3 supplemental treatments in a dual 3 × 3 Latin square design. Two animals were assigned to each treatment per period to determine the impact of salt level on dry matter intake, water intake, dry matter (**DM**) digestibility, liquid kinetics, and rumen fermentation. Prior to the initiation of each period of the Latin square, all heifers were weighed following a 16 hr shrink. Salt treatments consisted of 1) control, no salt (CON), 2) 0.05% of BW salt (LOW), and 3) 0.1% of BW salt (HIGH). A protein supplement of 50% cracked corn and 50% soybean meal fed at 0.3% of BW was mixed with salt treatments resulting in the salt and supplement combinations fed at 0.3%, 0.35%, and 0.4% for CON, LOW, and HIGH, respectively. Supplement/salt treatments were provided at 0800 and then after total consumption of supplement, the basal diet was offered. Diets were formulated to meet or exceed nutritional requirements for yearling heifers gaining 0.5 kg/d (NASEM, 2016). Chopped, low-quality, grass hay was used as the base ration and was provided daily at 120% of the average daily intake of the previous 3 d on an as-fed basis (Table 1). Average intakes were calculated on an as-fed basis (feed offered–feed refused). Before the start of the experiment, heifers were adapted to a salt-limited (25% salt) supplement for 14 d prior to the initiation of the trial.

**Table 1.** Composition of protein supplement and chopped grass hay fed to yearling heifers

Item	DM	TDN^1^	CP	NDF	ADF
Supplement^2^					
Period 1	90.4	83	36.1	9.4	7.1
Period 2	90.6	85	31.9	10.4	5.5
Period 3	90.7	85	30.1	9.9	5.1
Hay					
Period 1	93.6	55	7.5	65.4	42.9
Period 2	96.4	57	7.3	63.1	41.2
Period 3	95.1	57	7.4	64.2	41.5

^1^Total digestible nutrients, % = 88.9 – (0.779 × %ADF).

^2^Supplements were composed of 50% soybean meal and 50% corn; supplement nutrient composition was prior to the addition of salt.

### Sample collection and preparation

Each 22-d period included 14 d of diet adaptation, 6 d of sample collection (feed, orts, and feces), 1 d collection of rumen fluid samples for ruminal fermentation characteristics, and 1 d of complete ruminal evacuations. Feed and orts were collected daily prior to feeding (0700 hours) and subsamples were dried at 55 °C for 48 h to determine dry matter intake. All cattle were individually penned outdoors on a sloped corrugated concrete floor (14 × 6 m) containing a water trough mounted to a digital scale for measuring individual water intake. Daily fecal output was measured by manually collecting the total fecal output from the floor of each concrete pen prior to feeding. Daily fecal output was weighed and subsampled in duplicate. Fecal subsamples were dried at 55 °C for 96 h in a forced-air oven to calculate total fecal output on a DM basis. All dried feed, ort, and fecal samples were then ground to pass through a 1-mm screen using a Wiley mill. Daily water intake was measured by weighing daily disappearance of water corrected for evaporation (Wyffels et al., 2023).

Immediately following the 6-d sample collection period (day 21), each cow was intraruminally pulse-dosed with 286.25-mg/mL of a liquid marker (CrEDTA) in a 250-mL aqueous solution (71.5 g/dose; Udén et al., 1980) just before feeding (0800 hours). Rumen fluid samples were then obtained from multiple locations in the rumen/reticulum using a suction strainer (Raun and Burroughs, 1962) just before feeding and dosing (0 h) and at 4, 8, 12, 18, and 24 h after feeding to determine liquid kinetics, and rumen fermentation characteristics.

Ruminal contents were manually evacuated via the ruminal cannula on day 22, 5 h after feeding. Total contents were weighed, thoroughly mixed, and subsampled in duplicate (Van Soest, 1994). Ruminal subsamples were then weighed and dried in a forced-air oven at 55 °C for 96 h to determine liquid and DM fill.

### Laboratory methods

Feed and ort samples were analyzed for DM, CP, NDF, and ADF. Fecal samples were analyzed for DM and NDF for estimation of total-tract DM and NDF digestibility. Crude protein was analyzed using Leco combustion testing (Leco 828 Series, Leco Co.) to measure percent nitrogen which was then converted to crude protein (%N × 6.25). Neutral and acid detergent fiber were analyzed using an Ankom Fiber Analyzer (Ankom 200 Fiber Analyzer, Ankom Co.) in a sequential analysis.

Ruminal fluid samples were split into two 30 mL samples where one was measured for pH directly after extraction and the other was frozen immediately and stored at −20 °C and not thawed till the time of analysis (Nocek et al. 1987). Samples were analyzed for ammonia (NH_3_) concentrations using methods described by Sigma Technical Bulletin no. 640 (Chaney and Marbach, 1962; Horn and Squire, 1967; Weichselbaum et al., 1969). Individual VFA concentrations were analyzed using a gas chromatography procedure (Baumgardt, 1964; Byers, 1979; Fritz and Schenk, 1987). Chromium concentrations of rumen samples were analyzed by removing particulate matter via centrifuge (20 min at 4,000 × *g* at 20 °C) and using atomic absorption spectroscopy with a Perkin Elmer AAnalyst 300 equipped with an air/acetylene flame following the procedures described by Galyean (1997).

### Calculations

Forage intake, kg = Daily DM forage offered − daily DM forage refused

Supplement intake, kg = Daily DM supplement offered − daily DM supplement refused

Total intake, kg = (daily DM forage + daily DM supplement offered) − (daily DM forage + daily DM supplement refused)

Water intake, L = Daily water offered − daily water refused

DM digestibility, % = (Daily total intake − daily DM feces excreted) / (daily total intake)

NDF digestibility, % = (NDF of diet − NDF of Feces) / (NDF of diet)

Fluid dilution rate, %/h, was estimated by plotting the natural log of the ruminal Cr concentrations against sampling time. The slope of this log curve equals the fractional dilution rate (Galyean, 1997).

Ruminal fluid volume, L, was estimated by plotting the natural log of the ruminal Cr concentration against sampling time, extrapolating the ruminal Cr concentration at time zero, and dividing this value by the Cr dose administered (Galyean, 1997).

Fluid flow rate, L/h = Ruminal fluid volume × Fluid dilution rate (Galyean, 1997)

Total turnover time, h = (1/fluid dilution rate) × 100 (Galyean, 1997)

### Statistical analysis

The effects of salt level on intake, water consumption, and liquid kinetics were analyzed using an analysis of variance (ANOVA) with a generalized linear model for a replicated Latin square design including treatment, period, square, and animal as fixed effects. The effects of salt level on VFAs, pH, and ammonia were analyzed using ANOVA with generalized linear mixed models for a repeated measure analysis in a replicated Latin square design including treatment, period, square as fixed effects, and animal as a random intercept. Data were plotted and log-transformed if needed to satisfy assumptions of normality and homogeneity of variance. Preplanned orthogonal polynomial contrasts were used to determine linear and quadratic effects of treatment levels. Significance was determined at an alpha of <0.05 with tendencies considered between 0.05 and 0.10. All statistical analyses were performed in R (R Core Team, 2022).

## Results

Influence of salt level on intake and digestibility is listed in Table 2. Salt level had no influence on forage intake (*P* = 0.19), total intake (kg/d, *P* = 0.20), DM digestibility (*P* = 0.50), or NDF digestibility (*P* = 0.95). However, forage and total intake expressed as g/kg BW tended (*P* = 0.06) to decrease linearly with increasing levels of salt in the supplement. In contrast, water intake increased (*P* < 0.01) linearly with increasing salt levels ranging from 50.8 to 60.4 L/d.

**Table 2.** Effect of increasing salt levels on intake, digestibility, and rumen fill of yearling heifers consuming low-quality forages

	Salt levels^1^				*P*-values		
Item	CON	LOW	HIGH	SEM	TRT^2^	LIN^3^	QUAD^4^
Forage intake, kg	9.52	9.59	9.21	0.14	0.19	0.16	0.22
Supplement intake, kg	1.00	1.22	1.40	0.01			
Total intake, kg	10.53	10.62	10.23	0.14	0.20	0.19	0.21
Forage intake, g/kg BW	25.60	25.30	24.32	0.33	0.06	0.03	0.42
Supplement intake, g/kg BW	2.70	3.20	3.70	0.01			
Total intake, g/kg BW	28.30	28.01	27.02	0.33	0.06	0.03	0.42
Water intake, l	50.84	53.11	60.39	1.30	<0.01	<0.01	0.15
DM digestibility, %	56.29	56.32	54.98	0.88	0.50	0.32	0.54
NDF digestibility, %	51.21	51.26	50.64	1.54	0.95	0.80	0.86

^1^Salt levels include 1). CON, no salt, 2). LOW, 0.05% of BW, and 3). HIGH, 0.1% of BW.

^2^Treatment main effect.

^3^Linear preplanned contrast.

^4^Quadratic pre planned contrast.

DM fill tended to increase linearly with increasing levels of salt (*P* = 0.06; Table 3). In addition, liquid fill increased (*P* < 0.01) linearly with increasing levels of salt, with heifers in the high salt treatment having 17% greater liquid fill than control heifers. Fluid dilution rate decreased (*P = *0.04) linearly with increasing salt levels. Neither fluid flow rate nor ruminal liquid turnover time were influenced by salt levels in the diet (*P* ≥ 0.20) averaging 7.78 L/h and 9.95 h, respectively.

**Table 3.** Effect of increasing salt levels on fluid dilution rate, fluid flow rate, ruminal fluid volume, turnover time, liquid fill, and DM fill of yearling heifers consuming low-quality forages.

	Salt levels^1^				*P*-values		
Item	CON	LOW	HIGH	SEM	TRT^2^	LIN^3^	QUAD^4^
Fluid dilution rate, %/h	13.40	10.24	11.02	0.74	0.04	0.05	0.06
Fluid flow rate, L/h	8.36	7.17	7.82	0.49	0.29	0.46	0.17
Ruminal fluid volume, L	62.18	69.32	72.58	1.08	<0.01	<0.01	0.18
Turnover time, h	8.35	10.99	10.50	1.00	0.20	0.17	0.24
Liquid fill, L	62.67	69.87	73.16	1.09	<0.01	<0.01	0.18
DM fill, kg	10.80	11.53	12.12	0.33	0.06	0.02	0.86

^1^Salt levels include 1). CON, no salt, 2). LOW, 0.05% of BW, and 3). HIGH, 0.1% of BW.

^2^Treatment main effect.

^3^Linear preplanned contrast.

^4^Quadratic preplanned contrast.

**Figure 1.**
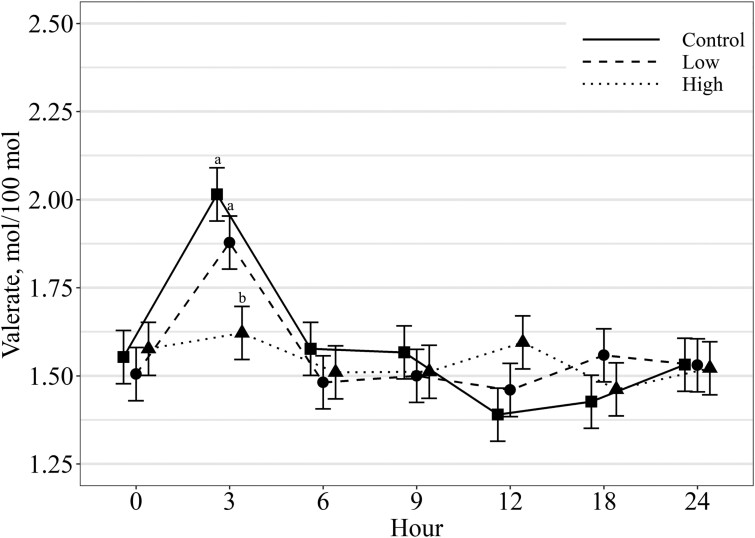
Effects of salt levels in supplement on concentration of valeric acid with an hour × treatment interaction (*P* = 0.01). Treatments include 1) CON, no salt, 2) LOW, 0.05% of BW, and 3) HIGH, 0.1% of BW. Means within hour that do not share a common letter differ (*P* < 0.05).

With the exception of Valerate, there were no observed treatment by sampling period (hour) interactions for all other rumen fermentation characteristics (*P* ≥ 0.28) with main effects presented in tabular form (Table 4). Ruminal pH and ammonia levels both decreased (*P* ≤ 0.01) linearly with increasing salt level. Total VFA concentrations and the acetate-to-propionate ratio were not influenced (*P* ≥ 0.59) by salt levels averaging 82.74 mM and 4.18, respectively. Acetate molar concentration increased (*P* < 0.01) linearly with increasing levels of salt. In contrast, propionate was not affected (0.94) by the treatment dietary levels of salt averaging 16.68 mol/100 mol. Isobutyrate and butyrate concentrations decreased (*P* < 0.01) linearly with increasing salt levels. Isovalerate was not influenced (*P* = 0.21) by increasing salt levels averaging 1.67 mol/100 mol. Valerate displayed a treatment × time interaction (*P* = 0.01) where treatment differences were only observed 3 h post-feeding with control and low salt having a higher molar concentration compared to the high salt treatment (Figure 1).

**Table 4.** Effect of salt levels on ruminal parameters of yearling heifers consuming low-quality forages

	Salt levels^1^				*P*-values			
Item	CON	LOW	HIGH	SEM	TRT^2^	TRT × HR^3^	LIN^4^	QUAD^5^
pH	6.90	6.87	6.76	0.04	<0.01	0.60	<0.01	0.14
Ammonia, mg/dL	4.42	3.92	3.53	0.34	0.01	0.88	<0.01	0.84
Acetate, mol/100 mol	68.60	69.23	69.62	0.33	<0.01	0.43	<0.01	0.67
Propionate, mol/100 mol	16.66	16.72	16.66	0.17	0.94	0.50	0.98	0.73
Isobutyrate, mol/100 mol	1.52	1.43	1.39	0.03	<0.01	0.86	<0.01	0.48
Butyrate, mol/100 mol	9.92	9.42	9.16	0.23	<0.01	0.28	<0.01	0.31
Isovalerate, mol/100 mol	1.73	1.65	1.63	0.06	0.21	0.80	0.10	0.57
A:P ratio^6^	4.14	4.19	4.21	0.06	0.59	0.54	0.33	0.74
Total VFAs, mM	84.16	82.55	81.51	3.91	0.84	0.75	0.56	0.94

^1^Salt levels include 1) CON, no salt, 2) LOW, 0.05% of BW, and 3) HIGH, 0.1% of BW.

^2^Treatment main effect.

^3^Treatment × Hour interaction.

^4^Linear preplanned contrast.

^5^Quadratic pre planned contrast.

^6^Acetate:propionate.

## Discussion

To date, literature discussing the influence of NaCl on forage digestibility is conflicting. Previous research has found no effect on nutrient digestion with high salt diets (Archer et al., 1952; Cardon, 1953; Nelson et al., 1955). However, Riggs et al. (1953) and Brandyberry et al. (1991) noted an increase in organic matter and protein digestibility, respectively, when cattle were offered a salt-limited protein supplement. Interestingly, intake expressed on a g/kg BW tended to decrease with increasing levels of salt, this displays an inefficiency since the goal of supplementation is to increase intake of available forage. Similarly, DM fill tended to increase with increasing levels of salt. The tendency for increasing DM fill and decreasing DM intake with increasing salt levels observed in this study suggests a decreased rate of digestion as a result of increased salt intake (Van Soest, 1994).

Increasing salt levels increased water intake and liquid fill. Increased water intake and liquid fill, in turn, can contribute to changes in liquid kinetics within the rumen which can be correlated with digestibility (Harvey et al., 1986). In contrast to our findings, previous research has concluded that rumen fluid dilution rate increased for both roughage and concentrate diets when hypertonic solutions (NaCl) were added directly to the rumen. Similarly, Wiedmeier et al. (1987) and Harvey et al. (1986) noted an increased liquid dilution rate of cattle fed elevated levels of NaCl. In addition, Rogers et al. (1979) reported total rumen outflow was increased when NaCl was intraruminally infused in steers that consumed a high roughage diet. This is consistent with later work which stated an increase in rumen fluid volume as well as rumen outflow with the addition of NaCl (Rogers and Davis, 1982). Other research has found an increased water intake in response to dietary salt (Croom Jr et al, 1982; Nack et al., 2021). Presumably, the increase in water intake in response to increased dietary salt is to maintain electrolyte homeostasis (Croom Jr et al., 1982).

The rumen microbiome is often a reflection of the feed constituents consumed by the animal (Cholewińska et al., 2020; Eberly et al., 2023). In our study, increasing levels of salt in the diet increased fluid dilution rate which may impact the microbial community and digestibility by affecting microbial generation time and loss of digestible substrate (Van Soest, 1994). Furthermore, consumption of salt may have a direct antimicrobial effect on the rumen microbiome which could result in reduced fiber digestion. Although not a component of this study, further research is warranted regarding supplemental salt intake on the ecology of the rumen microbiome.

Ruminal pH and ammonia levels both decreased with increasing salt levels, although pH levels were still within optimal levels for fermentation. Cellulolytic bacteria prefer a rumen pH above 6.0. When rumen pH falls below 6.0 cellulolytic bacteria are inhibited and fiber digestion is hindered (Stewart, 1977; Grant and Mertens, 1992; Van Soest, 1994). Croom et al. (1985) found ruminal pH of steers consuming 5% NaCl in their diet was 5.6 compared to 6.0 control diet. Brandyberry et al. (1991), Rogers et al. (1979); and Rogers and Davis (1982) recorded similar pH declines when steers fed high roughage diets were provided dietary salt. Additionally, ruminal ammonia also declined with increasing levels of dietary salt. However, the levels of ammonia in our study did not meet or exceed 5 mg/dL which has been suggested as a minimal value for optimal microbial metabolism (Satter and Slyter, 1974). This suggests that ruminal ammonia levels may not have been optimal for microbial metabolism and the addition of supplemental salt may contribute to further declines in available ammonia. However, when the increase in liquid volume is accounted for in this study, the actual total rumen ammonia pool did not differ among treatments.

Drops in pH can influence the rate of VFA absorption (Owens and Goetsch, 1988). While total VFA concentrations were not influenced by salt levels in this study, specific VFAs were impacted. Although we did see an increase in Acetate molar concentration with increased levels of salt, no differences were observed in propionate or the acetate:propionate ratio. In addition, the relative levels in acetate and the acetate:propionate ratio reported in this study are typical of low-quality forage-based diets (Carlisle et al. 2021). Our results agree with Harvey et al. (1986) who also reported that molar proportions of propionate, and the acetate: propionate ratio were not influenced by salt, however our study is specific to cattle consuming low-quality forages, which is representative of fall/winter grazing cattle on western rangelands and provided salt-limited supplements.

Past research evaluating salt-limited protein supplement intake has illustrated high degrees of variation within and between individual animals. Wyffels et al. (2020) supplemented 30% CP, 25% salt, canola-based pellets to winter grazing beef cattle and observed average intakes ranging from 0.5 to 1.5 kg/d and coefficient of variations (**CV**) of 60% to 150%. Similarly, Wyffels et al. (2021) supplemented 30% CP, 25% salt, and baked molasses blocks and observed average supplement intakes ranging from 0.5 to 0.9 kg/d and 150 to 250% CV. Although both studies report average daily supplement intake at or near the target, the CV associated with these values would suggest an intake range of 0.0 to 4.5 kg/d or 0.0 to 1.13 kg of salt/d. The salt levels in our study were 0.23 and 0.45 kg salt/d, with no variation in daily intake. Therefore, future research should consider evaluating higher levels of salt intake and variation in the frequency of delivery.

## Implications

Our results demonstrate that high salt diets alters rumen function by impacting digesta kinetics and ruminal fermentation. While salt may be a tool to assist in supplement intake regulation, the addition of dietary salt may also result in lower intakes and less efficient rumen fermentation of beef cattle consuming low-quality forages. In this study, salt was added to the diet up to 0.1% of BW, this is a conservative level and could be increased after acclimating the animal to see a more representative treatment of animals who overconsume salt-limited supplements. Additional research is needed evaluating higher levels of supplemental salt, as well as less frequent consumption of salt-containing supplements.

## Abbreviations

ADF: acid detergent fiber
BCS: body condition score
BW: body weight
CP: crude protein
CV: coefficient of variation
DM: dry matter
NDF: neutral detergent fiber
TDN: total digestible nutrients
